# Astrogliosis in aging and Parkinson’s disease dementia: a new clinical study with ^11^C-BU99008 PET

**DOI:** 10.1093/braincomms/fcac199

**Published:** 2022-08-18

**Authors:** Mohamed A Mohamed, Zhou Zeng, Marta Gennaro, Nicholas P Lao-Kaim, Jim F M Myers, Valeria Calsolaro, Grazia Daniela Femminella, Robin J Tyacke, Antonio Martin-Bastida, Roger N Gunn, David J Nutt, Paul Edison, Paola Piccini, Andreas-Antonios Roussakis

**Affiliations:** Department of Brain Sciences, Imperial College London, Hammersmith Hospital, London, UK; Department of Brain Sciences, Imperial College London, Hammersmith Hospital, London, UK; Xiangya Hospital of Central South University, Changsha, Hunan, P.R. China; Department of Brain Sciences, Imperial College London, Hammersmith Hospital, London, UK; Department of Brain Sciences, Imperial College London, Hammersmith Hospital, London, UK; Department of Brain Sciences, Imperial College London, Hammersmith Hospital, London, UK; Department of Brain Sciences, Imperial College London, Hammersmith Hospital, London, UK; Department of Brain Sciences, Imperial College London, Hammersmith Hospital, London, UK; Department of Translational Medical Sciences, University of Naples Federico II, Naples, Italy; Department of Brain Sciences, Imperial College London, Hammersmith Hospital, London, UK; Department of Brain Sciences, Imperial College London, Hammersmith Hospital, London, UK; Department of Neurology and Neurosciences, Clinica Universidad de Navarra, Pamplona-Madrid, Spain; Department of Brain Sciences, Imperial College London, Hammersmith Hospital, London, UK; Department of Brain Sciences, Imperial College London, Hammersmith Hospital, London, UK; Department of Brain Sciences, Imperial College London, Hammersmith Hospital, London, UK; Department of Brain Sciences, Imperial College London, Hammersmith Hospital, London, UK; Department of Brain Sciences, Imperial College London, Hammersmith Hospital, London, UK

**Keywords:** Parkinson’s disease dementia, PET, imidazoline binding sites, astrogliosis, aging

## Abstract

The role of astrogliosis in the pathology of brain aging and neurodegenerative diseases has recently drawn great attention. Imidazoline-2 binding sites represent a possible target to map the distribution of reactive astrocytes. In this study, we use ^11^C-BU99008, an imidazoline-2 binding sites-specific PET radioligand, to image reactive astrocytes *in vivo* in healthy controls and patients with established Parkinson’s disease dementia. Eighteen healthy controls (age: 45–78 years) and six patients with Parkinson’s disease dementia (age: 64–77 years) had one ^11^C-BU99008 PET-CT scan with arterial input function. All subjects underwent one 3 T MRI brain scan to facilitate the analysis of the PET data and to capture individual cerebral atrophy. Regional ^11^C-BU99008 volumes of distribution were calculated for each subject by the two-tissue compartmental modelling. Positive correlations between ^11^C-BU99008 volumes of distribution values and age were found for all tested regions across the brain within healthy controls (*P* < 0.05); furthermore, multiple regression indicated that aging affects ^11^C-BU99008 volumes of distribution values in a region-specific manner. Independent samples *t*-test indicated that there was no significant group difference in ^11^C-BU99008 volumes of distribution values between Parkinson’s disease dementia (*n* = 6; mean age = 71.97 ± 4.66 years) and older healthy controls (*n* = 9; mean age = 71.90 ± 5.51 years). Our data set shows that astrogliosis is common with aging in a region-specific manner. However, in this set-up, ^11^C-BU99008 PET cannot differentiate patients with Parkinson’s disease dementia from healthy controls of similar age.

## Teaser

This is an imaging study in Parkinson’s disease dementia with ^11^C-BU99008 PET (experimental radioligand developed to assess astrocytic distribution in the living brain). Mohamed M. and Zeng Z *et al*. report that astrogliosis is common with aging in a region-specific manner. Here, ^11^C-BU99008 PET does not differentiate these patients from healthy controls.

## Graphical Abstract

**Graphical abstract:**
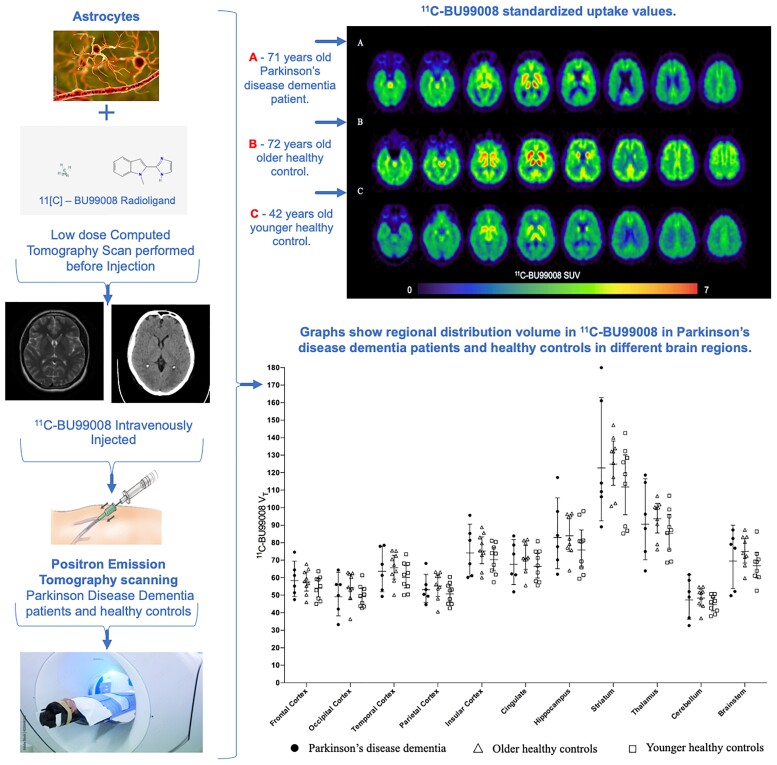

## Introduction

Astrocytes change morphology and functions in response to physiological aging, as well as to pathological stimuli, such as neurodegenerative diseases, infection, trauma, tumours, and ischaemia, through a process known as reactive astrocytosis (also termed as astrogliosis).^1,2^

Data from preclinical assays suggest that reactive astrocytes are involved in the progression of Parkinson’s disease,^3^ and Alzheimer’s^4^ diseases. Many experts in the field consider dementia in Parkinson’s disease (PDD) a sign of advanced Parkinson’s disease. However, the literature is inconclusive on the role of astrocytes in it. To our knowledge, no imaging study has yet examined the pattern of regional astrogliosis in PDD and its relation to brain aging. To date, only two PET radiotracers are available to image astrogliosis.^5^ Of these, the most well-known is ^11^C-deuterium-L-deprenyl (^11^C-DED), which binds irreversibly to the monoamine oxidase-B (MAO-B) and has been used to study Alzheimer’s disease, Creutzfeldt-Jakob disease, amyotrophic lateral sclerosis, and focal epilepsy.^6–9^ MAO-B is expressed on the outer mitochondrial membrane and is highly related with astrocytes. See relation with dopamine breakdown and the rationale of treating Parkinson’s disease with monoamine oxidate inhibitors (MAO-Is).

More recent work, using the relatively novel ligand, ^11^C-BU99008, which targets the imidazoline type-2 binding sites (I_2_BS) of the astrocyte mitochondrial membranes, enables the latter radiotracer as a promising biomarker of studying astrogliosis *in vivo.*^3,10,11^ The first publication with ^11^C-BU99008 PET in healthy controls (HCs), presents ^11^C-BU99008 as a tracer with a good global brain uptake and specific binding signal that is consistent with I_2_BS distribution known from pathology studies.^12^ More recently, ^11^C-BU99008 PET showed high levels of regional I_2_BS density in people with early Parkinson’s disease^13^ as well as in patients with established Alzheimer’s disease.^14,15^

The current study aims to investigate regional astrogliosis *in vivo* (with ^11^C-BU99008 PET) in patients with PDD and HCs. Secondarily, the study explores the role of aging on regional I_2_BS distribution.

## Materials and methods

### Participants

We included six patients with PDD between 64 and 77 years of age (mean age: 71.97 ± 4.66) and eighteen HCs, between 45–78 years of age (mean age: 61.94 ± 11.79), who did not have a family history of Parkinson’s disease. Patients were recruited from the Specialist Movement Disorders NHS Clinics, Charing Cross Hospital, Imperial College Healthcare NHS Trust, London, UK. All patients had established idiopathic Parkinson’s disease according to the UK Parkinson’s Disease Queen Square diagnostic criteria.^16^ The diagnosis of PDD was made using the clinical criteria of the Movement Disorders Society for probable PDD.^17^

HCs were recruited as discussed elsewhere.^13^ None of the HCs was taking medication with direct action on the CNS. All subjects (HCs and patients with PDD) were able to undergo MRI scanning. None of the patients was ever treated with MAO-Is. Severity of Parkinson’s disease symptoms was assessed using the modified Hoehn and Yahr staging scale^18^ and the Movement Disorders Society Unified Parkinson’s Disease Rating Scale (MDS-UPDRS).^19^ Global cognitive function was evaluated using the mini-mental state examination (MMSE),^20^ the Montreal cognitive assessment (MoCA)^21^ and the Parkinson’s disease Clinical Rating Scale (Parkinson’s disease-CRS).^22^ In this protocol, the following scores were deemed indicative for PDD: MMSE scores below 26, MoCA scores below 25, and Parkinson’s disease-CRS total scores ≤81. Read more about this approach elsewhere.^23^ Neuropsychiatric symptoms were assessed using the Neuropsychiatric Inventory (NPI).^24^ All patients were assessed clinically in the ‘off’ medication state. Clinical assessments took place at the NIHR Imperial Clinical Research Facility (London, UK) within a month prior to the imaging procedures.

Local Research Ethics Committee approvals were obtained before recruitment took place [National Research Ethics Services References: 17/NW/0009 (PDD subjects), 14/LO/1741 and 15/LO/1609 (HCs)]. All participants provided their written consent in accordance with the Declaration of Helsinki. Each sub-project was approved by the Health Research Authority, the Imperial Joint Research Office, and the UK Administration of Radioactive Substances Advisory Committee.

### Scanning procedures

All imaging was performed at Invicro LLC imaging facility (London, UK). ^11^C-BU99008 PET scans were acquired on a Siemens Biograph TruePoint HI-REZ 6 PET/CT system (Siemens Healthcare). In brief, a low-dose CT transmission scan (0.36 mSv) was performed prior to radioligand injection for attenuation-correction and anatomical localisation purposes.

^11^C-BU99008 was intravenously administered as a bolus injection (mean injected dose of 286.46 ± 28.56 MBq); dynamic emission data were acquired for 120 minutes after injection. The imaging data were reconstructed using a filtered back-projection algorithm (direct inversion Fourier transform; matrix size: 128 × 128, zoom: 2.6, 5 mm transaxial Gaussian filter, pixel size: 2 mm isotropic) and then binned into 29 frames. Before the administration of the radioligand, an arterial line was placed in the radial artery by experienced PET imaging physicians, following local anaesthesia and safety measures including Allen testing. Through this line, arterial blood was sampled continuously for the first 15 min of the scan. In addition, discrete blood samples were manually withdrawn at 5, 10, 15, 20, 25, 30, 40, 50, 60, 70, 80, 90, 100, 110, and 120 min after ^11^C-BU99008 venous injection.

T_1_-weighted structural MR brain images were acquired on a 3T Siemens Magnetom Trio system with a 32-channel head coil (MPRAGE; repetition time = 2300 ms, echo time = 2.98 ms, flip angle 9°, time to inversion = 900 ms, slice thickness 1 mm, matrix size 240 × 256 mm) to facilitate PET anatomical identification, and to assess the extent of regional volume loss (patients with PDD only).

### MRI data analysis

Subcortical nuclei volumes and cortical thickness were assessed from MRI data using FreeSurfer image analysis suite (version 6.0.0; http://surfer.nmr.mgh.harvard.edu) with a surface-based (cortical) and volume-based (subcortical) approach, as previously described.^25^ Briefly, the software executed skull-stripping, automated Talairach transformation, segmentation of the subcortical white matter and the deep grey matter volumetric structures, intensity normalisation, tessellation of the grey matter/white matter boundary, automated topology correction, intensity gradients based surface deformation to product grey/white matter and grey/cerebrospinal fluid surface model, and brain inflation. The volumes of subcortical nuclei were measured automatically from FreeSurfer’s output and normalized using total intracranial volume, also provided by FreeSurfer, to account for between-person variations in head size.

### PET image processing

PET and MR (MPRAGE) images and blood sample data were analysed using MIAKAT^TM^ software version 4.2.6 for academic purposes (Molecular Imaging and Kinetic Analysis Toolbox, Imanova Ltd),^26^ which was implemented on MATLAB (MathWorks Inc.), utilizing FSL 6.0 (FMRIB Image Analysis Group) and SPM 12 (Statistical Parametric Mapping, Wellcome Trust Centre for Neuroimaging) functions.

Structural MPRAGE images were segmented and rigid registered to the standard Montreal Neurological Institute (MNI) template. An MNI-based regional atlas (CIC Atlas v1.2; GlaxoSmithKline Clinical Imaging Centre) was non-linearly warped to the registered MPRAGE images to define regions of interest (ROI: whole brain, cortical regions, subcortical regions, cerebellum, brainstem, frontal cortex, occipital cortex, temporal cortex, parietal cortex, cingulate cortex, insula, hippocampus, striatum and thalamus).

Dynamic PET images were motion-corrected using rigid registration to reference frame 16. Signal-averaged (summed) images were created to define the co-registration parameters to corresponding MPRAGE images. ROI maps from the registered atlases were overlaid on the dynamic PET images to enable modelling of regional time-activity curves. Parent plasma input functions were calculated from the arterial blood samples. The whole-blood data were corrected for plasma and metabolite fractions and were interpolated with a triexponential function. These data were used as input function in a two-tissue compartment model with 5% fixed blood volume, which has been shown as the most suitable model to calculate ^11^C-BU99008 distribution volume (V_T_).^12^

To address potential bias from partial volume effects due to brain atrophy, we applied partial volume correction (PVC) on ^11^C-BU99008 PET images for patients with PDD and a subgroup within HCs (see definition of subgroups below) using the PETPVE12 toolbox.^27^ PVC was performed with the voxel-based Müller-Gartner compartmental model^28^ using the MNI-registered tissue segments derived as part of the main analysis pipeline. Corrected PET images were then generated from the co-registered dynamic series through an automated process, with a frame-by-frame approach. After that stage, corrected images were re-introduced on MIAKAT software. Respective ^11^C-BU99008 V_T_ values were calculated with and without PVC.

### Statistical analyses

All statistical analyses were performed with SPSS statistics (version 20); graphs were created using GraphPad Prism (version 6.0) for MAC OS X. Imaging and clinical numerical variables were tested for normality and equality of variances using Shapiro–Wilk and Levene’s tests, respectively. The level of significance was set at a = 0.05. Comparisons of means was made with independent samples *t*-test and Mann–Whitney U test, as appropriate. Within HCs, the association between age and regional ^11^C-BU99008 V_T_ values was sought by non-parametric Spearman’s rho. Benjamini-Hochberg correction procedure was applied.

To understand the effect of aging on astrogliosis, HCs were divided according to quartiles to: older healthy controls (OHCs) and younger healthy controls (YHCs). The differential effects of age on ^11^C-BU99008 V_T_ values across brain regions (cortical, subcortical areas and cerebellum) were compared via multiple linear regression (dependent variable: ^11^C-BU99008 V_T_, independent variables: region, age and age-by-region interaction; with cerebellum as a reference region). All assumptions for multiple regression were met. To assess differences of regional ^11^C-BU99008 V_T_ values, with or without PVC, between patients with PDD and OHC, independent samples *t*-tests were conducted separately for each ROI. Comparisons between groups on FreeSurfer MRI data were performed with Mann–Whitney U tests (two-tailed).

### Data availability

The authors confirm that the data supporting the findings of this study are available within the article and its supplementary material.

## Results

### Demographics and clinical characteristics

Table 1 summarizes the demographics and clinical characteristics of all participants. There was no significant difference in age between patients with PDD and OHC (*P* > 0.05). PDD patients were significantly older than the YHC (*P* < 0.001). Comparisons between PDD and HC groups refer to independent samples *t*-test (one-tailed).

**Table 1:** Characteristics of healthy controls and patients with PDD

	PDD	OHC	YHC
*n*	6	9	9
Age, (years)	71.97 ± 4.66	71.90 ± 5.51^ns^	51.97 ± 6.47***
Gender	3F:3M	2F:7M	1F:8M
PD duration (years)	7.37 ± 3.21	—	—
PDD duration (years)	1.05 ± 0.57	—	—
Hoehn and Yahr	3.33 ± 0.52	—	—
MDS-UPDRS Total	88.83 ± 26.78	—	—
MDS-UPDRS-Part I	10.83 ± 1.83	—	—
MDS-UPDRS-Part II	19.83 ± 10.85	—	—
MDS-UPDRS-Part III	51.00 ± 15.35	—	—
NPI total	2.83 ± 1.46	—	—
NPI-Hallucinations	0.50 ± 0.50	—	—
NPI-Depression	1.00 ± 1.00	—	—
NPI-Anxiety	0.67 ± 0.47	—	—
Daily LED_Total_ (mg)	387.17 ± 310.56	—	—
MMSE	21.17 ± 5.42	—	—
MoCA	15.33 ± 6.19	—	—
PD-CRS	52.83 ± 17.23	—	—

Values are shown as means ± SD. PDD scores calculated in ‘off’ medication state.

*** denotes significant at *P* < 0.001; ns, not statistically significant. Comparisons were made between PDD (*n* = 6) and OHC (*n* = 9) and between PDD and YHC (*n* = 9); independent samples *t*-test (one-tailed).

PDD, Parkinson’s disease dementia; OHC, older healthy controls; YHC, younger healthy controls; ns, not significant; F, female; M, male, MDS-UPDRs, Movement Disorders Society Unified Parkinson's disease rating scale; daily LED_Total_, daily total levodopa equivalent dose; MMSE, mini-mental state examination; MoCA, Montreal cognitive assessment; Parkinson’s disease-CRS, Parkinson’s disease Clinical Rating Scale.

### PET data analysis

**Figure 1:**
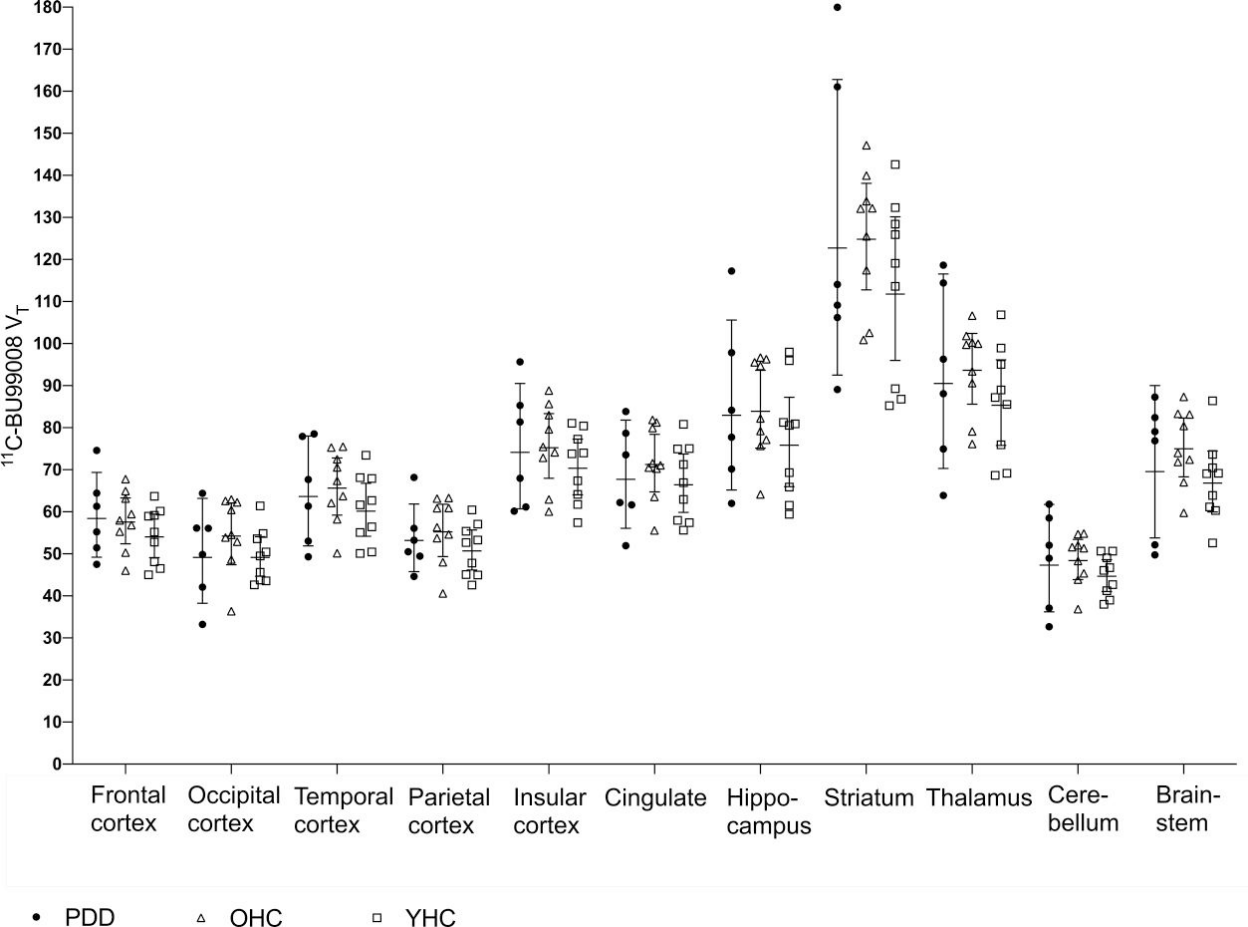
**Regional distribution volumes.** Graph shows regional ^11^C-BU99008 distribution volumes (V_T_) in PDD patients (*n* = 6) and healthy controls (OHC, *n* = 9; YHC, *n* = 9)V_T_, volume of distribution; PDD, Parkinson’s disease patients with dementia; OHC, older healthy controls; YHC, younger healthy controls.

**Figure 2:**
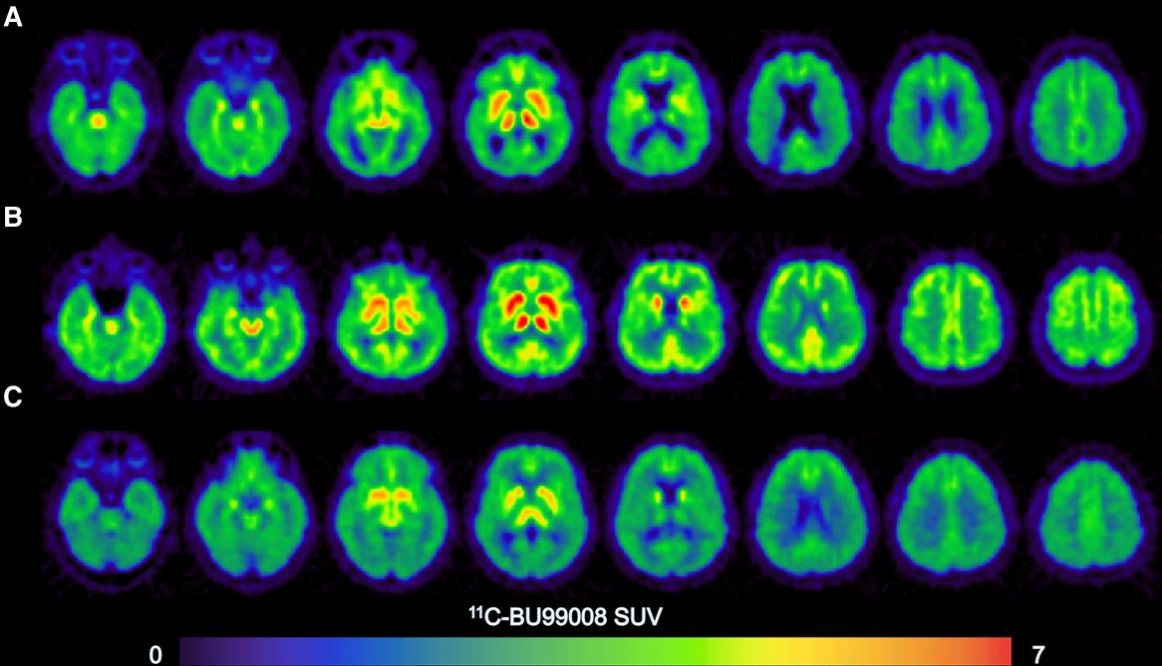
**^11^C-BU99008 standardised uptake values.** Representative images from three male subjects: a patient with PDD [(**A**); 71-year-old; MDS-UPDRS Total = 118, MoCA = 18], an older healthy control [(**B**); 72-year-old] and a younger healthy control [(**C**); 42-year-old].

The distribution of regional ^11^C-BU99008 binding for patients with PDD, OHC and YHC are presented in Table 2 and Figs. 1 and 2.

**Table 2:** ^11^C-BU99008 V_T_ uncorrected values (means) in patients with PDD and healthy controls

Region	PDD (*n* = 6)	HCs (*n* = 18)	OHC (*n* = 9)	YHC (*n* = 9)
Frontal cortex	59.09 ± 9.81	56.19 ± 6.85	57.97 ± 6.93	54.41 ± 6.67
Occipital cortex	50.29 ± 11.17	52.22 ± 7.86	54.95 ± 8.65	49.49 ± 6.31
Temporal cortex	64.64 ± 12.32	63.40 ± 8.58	66.16 ± 8.47	60.65 ± 8.23
Parietal cortex	53.68 ± 8.07	53.38 ± 7.12	55.71 ± 7.57	51.04 ± 6.18
Insular cortex	75.26 ± 14.37	73.33 ± 9.22	75.84 ± 9.73	70.81 ± 8.48
Hippocampus	84.85 ± 20.03	80.81 ± 13.13	84.62 ± 11.69	76.99 ± 14.04
Cingulate	68.64 ± 12.05	69.37 ± 8.91	71.74 ± 8.61	66.10 ± 9.06
Thalamus	92.71 ± 21.56	90.22 ± 12.23	94.19 ± 10.49	86.25 ± 13.13
Striatum	126.59 ± 35.55	119.72 ± 19.39	125.75 ± 15.95	113.69 ± 21.52
Cerebellum	48.52 ± 11.57	46.83 ± 7.26	48.76 ± 5.86	44.89 ± 4.85
Brainstem	71.26 ± 16.13	71.43 ± 9.87	75.46 ± 8.86	67.41 ± 9.59

Values are shown as means ± SD.

PDD, Parkinson’s disease dementia; HCs, healthy controls; OHC, older healthy controls; YHC, younger healthy controls.

#### Between-group comparisons

Independent samples *t*-test indicated no significant difference in regional ^11^C-BU99008 V_T_ values, between PDD and OHC, or between PDD and the entire HC group. Differences in regional ^11^C-BU99008 V_T_ were sought post-PVC (corrected); these results were consistent with those derived without PVC—see supplementary Table 1.

Both PDD and OHC groups had higher ^11^C-BU99008 V_T_ values in subcortical areas as compared with the cerebellum (*P* < 0.05). No significant difference was found between these groups in ^11^C-BU99008 V_T_ for either cortical or cerebellar values. Similarly, no regional difference was identified between PDD and OHC or between PDD and the entire HC group.

#### Correlations

There was no statistically significant correlation between regional ^11^C-BU99008 V_T_ values and clinical scores including motor (MDS-UPDRS) and cognitive (MMSE, MoCA, Parkinson’s disease-CRS) scores. Within all 18 HCs, Spearman’s rho testing revealed moderate-strong correlations between age and ^11^C-BU99008 V_T_ values in the frontal (r = +0.51, *P* < 0.05), occipital (*r* = +0.66, *P* < 0.01), temporal (*r* = +0.55, *P* < 0.01), parietal (*r* = +0.64, *P* < 0.01), insular (*r* = +0.49, *P* < 0.05), and cingulate cortices (*r* = +0.51, *P* < 0.05), hippocampus (*r* = +0.44, *P* < 0.05), striatum (*r* = +0.47, *P* < 0.05), thalamus (*r* = +0.57, *P* < 0.01), cerebellum (*r* = +0.54, *P* < 0.01) and brainstem (*r* = +0.58, *P* < 0.01).

#### Age effects

**Figure 3:**
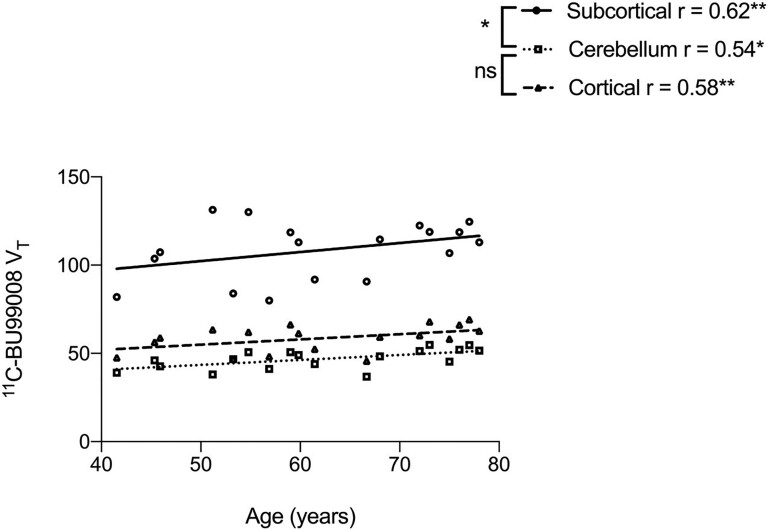
**Correlations.** Spearman’s correlations (two-tailed) between age and ^11^C-BU99008 V_T_ values in subcortical regions, the cerebellum, and cortical regions in HCs (*n* = 18). Age and ^11^C-BU99008 V_T_ values showed strong correlations for subcortical regions (r = +0.62, *P* = 0.004), the cerebellum (r = +0.54; *P* = 0.02), and cortical regions (r = +0.58, *P* = 0.009). * denotes significant at *P* < 0.05; ** denotes significant at *P* < 0.01; V_T_, volume of distribution.

A significant age-by-region interaction was observed in HCs, revealing differential age effects on ^11^C-BU99008 V_T_ values for each region. As shown in Fig. 3, the effect of aging on ^11^C-BU99008 V_T_ was more prominent in subcortical regions, compared to the cerebellum (*P* < 0.05). ^11^C-BU99008 V_T_ values were significantly higher in subcortical and cortical regions than in the cerebellum (*P* < 0.001), irrespective of age. We found no significant effect of age on ^11^C-BU99008 V_T_ when values were collapsed across regions. The results of multiple linear regression are presented in Table 3.

**Table 3:** Result of multiple linear regression analysis in healthy controls (*n* = 18) with interaction term

Variables		Adj. R^2^	ΔR^2^	B	SE	β	significance
		0.908	0.917				
Age				0.33	0.32	0.07	ns
^a^Region							
	Cortical			37.55	5.20	0.35	***
	Subcortical			114.6	5.20	1.06	***
Age × ^a^region							
	Cortical			0.31	0.45	0.04	ns
	Subcortical			1.03	0.45	0.13	*

*denotes significant at *P* < 0.05; *** denotes significant at *P* < 0.001.

Adj. R^2^, adjusted R square; B, unstandardized coefficients; β, standardized coefficients beta; ΔR^2^, R square change; ns, not statistically significant; Referent region for ^a^region: cerebellum; SE, standard error.

### MRI data analysis

Patients with PDD had significant volume losses as compared with OHC in several brain regions. Comparisons refer to Mann–Whitney U test (two-tailed) between PDD and OHC or between OCH and YHC. Volumetric analyses showed a significant reduction in the left hippocampus (PDD: 3.24 ± 0.29 cm^3^, OHC: 3.91 ± 0.17 cm^3^, two-tailed Mann–Whitney U test, U = 0, *P* < 0.05) and right hippocampus (PDD: 3.47 ± 0.33 cm^3^, OHC: 4.13 ± 0.32 cm^3^, two-tailed Mann–Whitney U test, U = 1, *P* < 0.05), right putamen (PDD: 3.63 ± 0.63 cm^3^, OHC: 4.47 ± 0.34 cm^3^, two-tailed Mann–Whitney U test, U = 2, *P* < 0.05), left amygdala (PDD: 1.49 ± 0.23 cm^3^, OHC: 1.80 ± 0.30 cm^3^, two-tailed Mann–Whitney U test, U = 5, *P* < 0.05), and right nucleus accumbens (PDD: 0.38 ± 0.04 cm^3^, OHC: 0.47 ± 0.06 cm^3^, two-tailed Mann–Whitney U test, U = 3, *P* < 0.05). PDD data refer to direct comparison with data from the OHC group (*P* < 0.05). Significant volume losses were also observed in the brainstem of the OHC group as compared with YHC (OHC: 20.37 ± 1.67 cm^3^, YHC: 23.10 ± 2.96 cm^3^, two-tailed Mann–Whitney U test, U = 15, *P* < 0.05). See more in supplementary Table 2.

## Discussion

This is the first PET imaging study with ^11^C-BU99008 to explore the extent of astrogliosis in PDD. We found that I_2_BS density relates to old age in a region-dependent manner as reflected by data from HCs. However, I_2_BS density values did not differentiate patients with PDD from healthy subjects of similar age.

In HC groups, I_2_BS density values, across the brain, correlated positively with age, suggesting that astrogliosis is more common as individuals get older. This finding is in line with ^11^C-DED PET human studies in HCs—reference to MAO-B density increases.^29,30^ This point is supported by evidence stemming from post-mortem and animal studies on the astrocytic glial fibrillary acidic protein (GFAP).^30–32^ The GFAP helps astrocytes maintain their structural integrity and to achieve (in the reactive state) cell movement. The GFAP is believed to increase in the cytoplasm of astrocytes, when dramatic changes in their shape are required. Alterations in reactive astrocytes of humans, non-human primates and rats, implicate GFAP as a significant site for research.^31–33^ Although, to our knowledge, there is no GFAP-specific PET radioligand for human use. As astrocytic I_2_BS density relates to high GFAP expression,^34,35^ we suggest that brain imaging with ^11^C-BU99008 PET represents a valuable marker of reactive astrocytosis *in vivo.*^3,12,13^

Our HC group data demonstrate that aging affects global I_2_BS density albeit with some preference to the brain regions known from preclinical and pathology studies^32,36,37^ to be most vulnerable to degeneration. Our PDD data support this view suggesting that astrogliosis in a region-specific manner reflects selective vulnerability to progressive neuronal loss. Future work will be required to explore the impact of aging-related astrogliosis on neuronal networks, and the chronology of cognitive impairment and motor dysfunction in Parkinson’s disease.

Our findings describe similar levels of cerebral I_2_BS binding between the PDD and OHC groups. Unfortunately, external evidence from studies on astrogliosis within PDD is limited to further support this finding. Two post-mortem studies have shown the presence of reactive astrocytes in the substantia nigra of Parkinson’s disease patients, some of whom were demented,^38,39^ whereas others could not demonstrate a robust difference in astrogliosis patterns between demented/non-demented Parkinson’s disease patients and HCs.^40,41^ In a recent study, ^11^C-BU99008 PET data from patients with early Parkinson’s disease showed high global I_2_BS binding as compared with HCs.^13^ Under the same imaging protocol, global I_2_BS binding was relatively low in patients with moderate/advanced Parkinson’s disease, as compared with data from both early stage Parkinson’s disease and HCs. In this PET study, lower ^11^C-BU99008 binding correlated with worse global cognitive scores (MoCA assessments) in moderate/advanced Parkinson’s disease patients^13^ without distinguishing between demented and non-demented Parkinson’s disease patients.

In context with previous work,^42^  ^11^C-BU9008 PET data^13^ suggest that astrogliosis follows a non-linear trend in the course of Parkinson’s disease. By reviewing our data together with evidence from other reports in the field, we propose, that the extent of astrogliosis in non-demented patients might reach a relatively high level in early Parkinson’s disease.^13^ Nevertheless, in advanced Parkinson’s, with or without dementia, astrogliosis markers could measure within normal or mildly lower levels. This makes a point for a difference between ‘severe motor disease’ and ‘advanced Parkinson’s disease with dementia’.

A non-linear pattern of astrogliosis within PDD would likely be propagated by astrocytic α-synuclein accumulation.^41,43^ Indeed, previous cell culture studies in early Parkinson’s disease demonstrated that astrocytes can clear α-synuclein deposits from damaged neurons.^44,45^ However, pathology processes involve excessive astrocytic α-synuclein accumulation in the course of Parkinson’s disease. These processes suppress innate protective astrocytic responses and enable further neuronal damage.^41,43^ In a recent post-mortem study in advanced Parkinson’s disease,^43^ researchers found a negative correlation between levels of α-synuclein accumulation and markers of astrogliosis, including the GFAP. This point suggests that astrogliosis in regions of relevance for Parkinson’s disease, may steadily decline after a plateau of significant elevation. See evidence from markers of reactive astrocytosis in early Parkinson’s disease together with our PDD data.

Beyond Parkinson’s disease, a trend for steady decline of astrogliosis is supported by ^11^C-DED PET data (targeting the MAO-B) in carriers of autosomal dominant Alzheimer’s disease.^46^ Within a preserved region, it is possible, that astrocytes (in Parkinson’s disease as well as autosomal dominant Alzheimer’s disease), partly, retain the mechanism of beneficial α-synuclein clearance—reference to comparable ^11^C-BU99008 V_T_ values in HC groups. However, in patients with marked regional degeneration (such as in advanced Parkinson’s disease), astrocytic clearance may be increasingly impaired due to severe reductions in neuronal cell count. Our PET and MRI data support this hypothesis and propose that a significant part of I_2_BS measures in PDD is due to aging-related processes.

We are aware of potential methodological issues in the current work, including the size of our PDD group and the explorative nature of the study. At this point, we would like to make some disclosures to clarify our viewpoint and justify our approach. By reviewing the literature, we decided to exclude patients with previous lifetime exposure to MAO-Is. We understand this as a very important exclusion criterion for imaging the I_2_BS, especially with a relatively novel tracer. In the clinical environment, many patients with Parkinson’s disease are chronically prescribed irreversible MAO-Is, such as rasagiline. It would have been possible to recruit patients who routinely take these medicines and instruct them to temporarily withdraw for the purpose of this study. However, we deemed it important to have minimal risks from exogenous influences on our PET data and so excluded previous lifetime exposure.

We acknowledge that long scanning procedures are challenging for clinical research purposes. Recruiting Parkinson’s disease patients with confirmed dementia can be laborious, especially when the target is a group of patients who are able to provide informed consent, complete questionnaires, and execute high function tasks. More relevantly for the current work, eligible subjects were challenged to comply with the demands of long and invasive scanning procedures. For the present study, this refers to the difficulty to recruit patients with Parkinson’s disease with no previous exposure to MAO-Is, the difficulty to identify participants eligible for arterial cannulation (exclusion associated with commonly prescribed anticoagulants that form a contraindication for arterial cannulation), and capable of undergoing MR imaging. These points are made to explain the realistic approach for a small sample size for the disease under study.

We would like to state the lack of consensus on optimal PVC modelling and analysing ^11^C-BU99008 dynamic data. There may be a heterogeneity of ^11^C-BU9008 affinity for the I_2_BS in the general population. In a recent post-mortem report in Alzheimer’s disease, researchers detected multiple binding sites for BU9008, and categorized their cases as super-high-affinity, high affinity and low affinity subjects.^47^ Testing for affinity status prior to scanning was not available at the time we conducted this study; to our knowledge, this parameter was not considered in the recently published ^11^C-BU99008 PET protocols.

In this study, we recruited demented Parkinson’s disease patients who had capacity to provide informed consent excluding demented patients who would have very low to zero scores to ensure patient engagement, protocol adherence and to avoid floor effects. Here, PDD was confirmed based on clinical criteria (MDS criteria for probable PDD). We agree that validation of PDD diagnosis with clinical scales is challenging. The Parkinson’s disease-CRS cut-off point of 81/134 on its own is probably higher than what would in reality constitute PDD. However, the currently published suggestions for PDD cut-offs are rather inconsistent themselves.^23^ In our study, all patients had Parkinson’s disease CRS scores below 73.5/134, which meets the cut-off proposed most recently.^23^ Scores are also influenced by educational and socioeconomic parameters. To avoid incorrectly excluding patients, we decided to take a more liberal threshold for this scale (= 81), and cite a review article that discusses these figures in depth.^23^ We acknowledge that some of our recruited patients may demonstrate scores similar to patients with mild cognitive impairment features and consider this a methodological limitation.

Our group of patients is theoretically not representing most patients of the general PDD population. We appreciate that there is no Parkinson’s disease-control group i.e. a group of non-demented Parkinson’s disease patients who would be selected to match for age our PDD group. However, our design is that of an observational study to explore astrogliosis in an otherwise heterogeneous PDD group (reference to variability in motor severity scores and disease duration parameters). We believe that for the purposes of studying PDD with ^11^C-BU99008 PET imaging, our data set is appropriate and a base for discussion and future work in the field.

In this regard, we regard published methodology for astrocytic markers a subject of debate and an area for further exploration. We believe that conclusions on the value of ^11^C-BU99008 PET can only be driven after careful consideration of all available evidence with input from future imaging studies.

## Conclusion

^11^C-BU99008 PET is a sensitive biomarker for the study of astrogliosis *in vivo*. Our results suggest that astrogliosis is commonplace and highly related to aging in a region-specific manner. The design of our study provides an important resource for future imaging studies on the role of reactive astrocytes in cognitive decline and related neurodegenerative diseases.

## Supplementary Material

fcac199_Supplementary_Data

## Abbreviations

GFAP =: glial fibrillary acidic protein
I_2_BS =: imidazoline 2 binding sites
LED =: levodopa equivalent dose V_T_: volume of distribution
MDS-UPDRS =: Movement Disorders Society Unified Parkinson’s Disease Rating Scale
MMSE =: Mini-Mental State Examination
MoCA =: Montreal Cognitive Assessment
NPI =: Neuropsychological Inventory
PD-CRS =: Parkinson’s disease Clinical Rating Scale
PDD =: Parkinson’s disease dementia
PVC =: partial volume correction
