# Supplementary material for: Astrogliosis in aging and Parkinson’s disease dementia: a new clinical study with 11C-BU99008 PET
Source: Brain Commun. 2022 Aug 18;4(5):fcac199. doi: 10.1093/braincomms/fcac199 (PMC9445175; doi:10.1093/braincomms/fcac199)
Supplement: fcac199_Supplementary_Data [file fcac199_supplementary_data.pdf]

Supplementary material

Supplementary Table 1

^{11}C-BU99008 V_T values (means) with partial volume correction in patients with PDD, and OHC

Region	PDD (n=6)	OHC (n=9)
Frontal cortex	119.09 ± 20.78	105.83 ± 13.40
Occipital cortex	84.70 ± 20.48	89.10 ± 13.39
Temporal cortex	115.33 ± 24.55	107.48 ± 15.27
Parietal cortex	100.35 ± 15.38	97.71 ± 12.20
Insular cortex	119.09 ± 21.06	112.16 ± 16.49
Hippocampus	125.28 ± 20.58	121.44 ± 20.09
Cingulate	127.47 ± 24.97	119.90 ± 17.39
Thalamus	151.73 ± 29.66	143.88 ± 17.96
Striatum	256.34 ± 51.88	231.65 ± 37.83
Cerebellum	63.17 ± 19.35	62.46 ± 7.29
Brainstem	114.82 ± 34.09	114.08 ± 14.74

Values are shown as means ± SD.

PDD: Parkinson’s disease dementia; OHC: older healthy controls

Supplementary Table 2

Volumetric MRI data (means) in patients with PDD, and healthy controls

Brain region	PDD (n=6)	OHC (n=9)	YHC (n=9)	PDD versus OHC (p value)	OHC versus YHC (p value)
Thalamus_lh	6.20±0.42	6.31±0.67	6.96±0.75	0.720 ns	0.077 ns
Caudate_lh	3.05±0.36	3.28±0.29	3.34±0.37	0.207 ns	0.712 ns
Putamen_lh	3.78±0.62	4.28±0.44	4.66±0.54	0.103 ns	0.125 ns
Pallidum_lh	1.79±0.33	1.95±0.21	1.99±0.18	0.289 ns	0.666 ns
Amygdala_lh	1.28±0.24	1.56±0.15	1.58±0.16	0.019*	0.798 ns
Accumbens_lh	0.35±0.07	0.40±0.09	0.48±0.06	0.244 ns	0.046*
Hippocampus_lh	3.24±0.29	3.91±0.17	4.00±0.43	0.000***	0.566 ns
Thalamus_rh	5.89±0.44	6.41±0.58	7.01±0.73	0.094 ns	0.078 ns
Caudate_rh	3.10±0.45	3.44±0.36	3.45±0.34	0.136 ns	0.969 ns
Putamen_rh	3.63±0.63	4.47±0.34	4.56±0.62	0.008**	0.700 ns
Pallidum_rh	1.97±0.51	1.81±0.22	1.98±0.29	0.435 ns	0.189 ns
Hippocampus_rh	3.47±0.33	4.13±0.32	4.12±0.38	0.003**	0.970 ns
Amygdala_rh	1.49±0.23	1.80±0.30	1.80±0.14	0.054 ns	0.990 ns
Accumbens_rh	0.38±0.04	0.47±0.06	0.48±0.04	0.007**	0.486 ns
Brainstem	20.71±2.83	20.46±1.67	23.10±2.96	0.840 ns	0.039*

Values are means ± SD. Subcortical nuclei volumes are in cm^3. Volumes are normalised for total intracranial volume.

* denotes significant at $p<0.05$; ** denotes significant at $p<0.01$; *** denotes significant at $p<0.001$; ns: not statistically significant. Comparisons in volumes were made with Mann-Whitney U tests (two-tailed) between patients with PDD and OHC / between OHC and YHC.

PDD: Parkinson’s disease dementia; OHC: older healthy controls; YHC: younger healthy controls; lh: left hemisphere; rh: right hemisphere
